# Self-Reported Executive Functioning in Everyday Life in Parkinson's Disease after Three Months of Subthalamic Deep Brain Stimulation

**DOI:** 10.1155/2015/461453

**Published:** 2015-06-18

**Authors:** Uyen Ha Gia Pham, Stein Andersson, Mathias Toft, Are Hugo Pripp, Ane Eidahl Konglund, Espen Dietrichs, Ulrik Fredrik Malt, Inger Marie Skogseid, Ira Ronit Hebolt Haraldsen, Anne-Kristin Solbakk

**Affiliations:** ^1^Department of Psychosomatic Medicine, Oslo University Hospital-Rikshospitalet, 0424 Oslo, Norway; ^2^Institute of Clinical Medicine, University of Oslo, 0316 Oslo, Norway; ^3^Department of Psychology, University of Oslo, 0316 Oslo, Norway; ^4^Department of Neurology, Oslo University Hospital-Rikshospitalet, 0424 Oslo, Norway; ^5^Oslo Centre of Biostatistics and Epidemiology, Oslo University Hospital, 0424 Oslo, Norway; ^6^Department of Neurosurgery, Oslo University Hospital-Rikshospitalet, 0424 Oslo, Norway; ^7^Department of Neuropsychology, Helgeland Hospital, 8607 Mosjøen, Norway

## Abstract

*Objective*. Studies on the effect of subthalamic deep brain stimulation (STN-DBS) on executive functioning in Parkinson's disease (PD) are still controversial. In this study we compared self-reported daily executive functioning in PD patients before and after three months of STN-DBS. We also examined whether executive functioning in everyday life was associated with motor symptoms, apathy, and psychiatric symptoms. *Method*. 40 PD patients were examined with the Behavior Rating Inventory of Executive Function-Adult Version (BRIEF-A), the Symptom Checklist 90-Revised (SCL-90-R), and the Apathy Evaluation Scale (AES-S). *Results*. PD patients reported significant improvement in daily life executive functioning after 3 months of STN-DBS. Anxiety scores significantly declined, while other psychiatric symptoms remained unchanged. The improvement of self-reported executive functioning did not correlate with motor improvement after STN-DBS. Apathy scores remained unchanged after surgery. Only preoperative depressed mood had predictive value to the improvement of executive function and appears to prevent potentially favorable outcomes from STN-DBS on some aspects of executive function. *Conclusion*. PD patients being screened for STN-DBS surgery should be evaluated with regard to self-reported executive functioning. Depressive symptoms in presurgical PD patients should be treated. Complementary information about daily life executive functioning in PD patients might enhance further treatment planning of STN-DBS.

## 1. Introduction

Parkinson's disease (PD) is associated with cognitive deficits in a substantial number of patients. The severity of cognitive impairment can range from mild difficulties to global decline [1]. In PD, deficits in executive functioning are among the most profound cognitive impairments. Deficits are particularly observed in later stages of the disease but can also occur in earlier stages [2] and are associated with dysfunction of the dorsolateral prefrontal-striatal circuit [3]. Executive functions refer to higher-order cognitive functions involved in the control and regulation of cognitive processes needed for goal-directed behavior [4]. A frequent clinical expression of executive impairment in PD is a reduced ability to plan, organize, initiate, and sequence purposeful behavior. Executive deficits are related to disease characteristics and neuropsychiatric symptoms [5].

As a neuropsychiatric syndrome, apathy can frequently occur in PD [6]. Apathy is defined as the simultaneous diminution in all three aspects of goal-directed behavior, the emotional, the behavioral, and the cognitive domains. A common clinical manifestation of both executive dysfunction and apathy is lack of goal-directed behavior. Previous studies have reported that executive dysfunction is positively related to apathy in PD patients [7, 8]. The anatomic correlations of apathy in PD have not been fully elucidated, but research suggests that disruption to frontostriatal circuits and the mesolimbic system may play a significant role [9].

Among the non-motor symptoms associated with PD, psychiatric complaints are also common and can significantly impact the patients' quality of life [10]. Somatization, depression, anxiety, and obsessive-compulsive symptoms are the most prevalent self-reported psychiatric symptoms [11].

Deep brain stimulation of the subthalamic nucleus (STN-DBS) is a well-established therapeutic method to relieve motor symptoms for patients with advanced PD. However, some PD patients can experience neuropsychiatric changes after DBS [12]. Apathy is described following STN-DBS [13, 14].

With regard to executive function after STN-DBS, some studies concluded that aspects of executive function improved [15, 16], while other studies reported impaired executive functioning following STN-DBS [17, 18].

Beyond studies using performance-based cognitive tests, the authors are not aware of studies exploring how PD patients experience their executive functioning in everyday life before and after initiation of STN-DBS. Evaluation of PD patients' own perception of their daily executive functioning could be helpful in reconciling contradictory findings regarding executive functioning after STN-DBS. Therefore, the main goal of this study was to examine how PD patients perceive their executive functioning in everyday life after initiation of STN-DBS treatment compared to their presurgery functioning. We also explored whether motor symptoms, psychiatric symptoms, or apathy was associated with self-reported executive functioning in this patient group.

## 2. Patients and Methods

Patients' characteristics and methods have previously been published by Pham et al. [19]. The present study included 40 patients with PD who received STN-DBS at Oslo University Hospital-Rikshospitalet between 2009 and 2012. To be included in the study, the following criteria had to be met: (a) diagnosis of PD for more than five years, (b) MDS-Unified Parkinson's Disease Rating Scale (UPDRS-III) motor sub-score > 20 points, and (c) severe motor problems that were not controlled by optimized medical treatment. Exclusion criteria included (a) presence of severe cognitive impairment, defined as a total score of <130 on the Mattis Dementia Rating Scale, (b) severe untreated psychiatric illness, such as current major depression, psychosis, or hypomania/mania, and (c) language problems (Table 1).

Neurological assessments included neurological examination, Hoehn and Yahr staging of disease severity, and levodopa test comparing the MDS-UPDRS-III motor scores in the off-medication and on-medication states and were all conducted preoperatively and at the postoperative evaluation after three months of continuous STN-DBS.

Cerebral MRI scanning was performed before the CRW stereotactic frame was mounted for stereotactic 3D CT imaging. CT images were fused with the MRI, and the trajectories and surgical targets were then planned. Target locations for the permanently implanted electrodes were further refined intraoperatively using a combination of microelectrode recordings and intraoperative test stimulation. The electrodes were fixed to the skull, the extension leads were connected, and a neurostimulator was implanted.

### 2.1. Questionnaires

The Behavior Rating Inventory of Executive Function-Adult Version (BRIEF-A) is a 75-item self-report questionnaire exploring everyday behaviors in which executive functions are implicated. The BRIEF-A has previously been used in studies assessing daily life executive functioning in patients with a variety of clinical conditions such as traumatic brain injuries, mild cognitive impairment, Attention Deficit Hyperactivity Disorder, eating disorders, and patients with prefrontal lesions. The BRIEF-A has been used in evaluating perceived executive dysfunction in Parkinson's disease in two recent studies regarding executive functioning in PD [20, 21].

The questionnaire is completed by the patients and provides a subjective overview of cognitive difficulties during completion of everyday tasks [22]. It is designed to capture higher-order cognitive processes required to properly engage in real-world, goal-directed behaviors. The BRIEF-A consists of nine subscales (Inhibit, Shift, Emotional Control, Self-Monitor, Initiate, Working Memory, Plan/Organize, Task Monitor, and Organization of Materials), two index scales (Metacognition Index and Behavioral Regulation Index), and a general composite score, the Global Executive Composite (GEC). The Behavioral Regulation Index (BRI) is composed of the Inhibit, Shift, Emotional Control, and Self-Monitor scales and measures the ability to appropriately regulate behavioral and emotional responses. The Metacognition Index (MI) is composed of the Initiate, Working Memory, Plan/Organize, Task Monitor, and Organization of Materials scales and measures the ability to actively organize information and solve problems as they are encountered during completion of complex tasks. The skills reflected in the BRI are believed to support the ability to engage in appropriate metacognitive problem solving as measured by the MI. *T*-scores used for comparison are based on the normative sample of 2250 BRIEF-A reports [22]. Higher scores reflect greater difficulties, with *T*-scores above 65 considered as clinically significant.

The BRIEF-A also includes three validity scales. Scores on the Negativity scale measure to what extent the respondent answered selected items in an unusually negative manner, whereas scores on the Inconsistency scale indicate to what extent the respondent answered similar questions in an inconsistent manner. Scores on the Infrequency scale indicate whether the respondents tended to report highly unusual symptoms. A raw score of six or higher on the Negativity scale, three or higher on the Infrequency scale, and eight or higher on the Inconsistency scale indicates possible invalid protocols.

The Symptom Checklist 90-Revised (SCL-90-R) [23] is a self-assessment of psychiatric symptoms. The 90 items are scored on a five-point Likert scale, ranging from not at all (0) to extremely (4). The nine primary symptom dimensions are Somatization (SOM); Obsessive-Compulsive (O-C); Interpersonal Sensitivity (I-S); Depression (DEP); Anxiety (ANX); Hostility (HOS); Phobic Anxiety (PHOB); Paranoid Ideation (PAR); and Psychoticism (PSY). In addition, the SCL-90-R provides a global index of psychiatric symptoms, the Global Severity Index (GSI). The GSI represents the mean score of all 90 items of the SCL-90-R, with a score of 1 or more indicating probable psychiatric case [24]. The normative sample used for comparison of SCL-90-R results consisted of 466 men and 507 women representative of the adult population in Norway [24].

The Apathy Evaluation Scale (AES) is a rating scale consisting of 18 items. The self-rated version of the AES was administrated (AES-S) [25]. Consistent with the operational definition of apathy, there are three types of items: each item is primarily an index of overt goal-directed behavior, goal-related cognition, or goal-related emotional response. Items are rated on a four-point Likert scale ranging from 1 to 4. Sum scores range from 18 to 72. Higher scores indicate greater levels of apathy. As per current recommendation, a cut-off score of 38 was applied [25] (see Table 3).

### 2.2. Statistical Methods

All statistical analyses were conducted with PSAW Statistics 18. Demographic and clinical data were described as mean (SD) or number of patients. One-sample Student's *t*-tests were performed to compare normative data and data from the patient group (BRIEF-A, SCL-90-R). Comparisons between baseline and three months postsurgery scores were performed using paired sample Student's *t*-tests. The associations between measures were analyzed with Pearson's correlation coefficients. Stepwise multiple regression analysis was conducted to explore possible predictors to the change in BRIEF-A scores. Results were reported with a significance level of *p* ≤ 0.05. Of the 40 patients, five had more than 20% missing scores on the BRIEF-A at three-month follow-up, leaving 35 valid TCI protocols for pairwise comparison. At the end of the inclusion period, 36 patients had completed SCL-90-R both preoperatively and 35 patients at the three-month follow-up. Two patients preoperatively and six patients at three-month follow-up had more than 20% missing on the AES. There were relatively many variables being explored in this study. We did not attempt to correct for the level of significance for multiple comparisons. However, we were aware of the possibility of type I errors. The effect size of changes between preoperative and at three-month follow-up were assessed with Cohen's *d*. Cohen's *d* can define effect sizes as small (*d* = 0.2), medium (*d* = 0.5), and large (*d* = 0.8).

## 3. Results

### 3.1. Motor Outcome

The average “off” MDS-UPDRS III score at baseline was 48.9 and decreased to 18.8 after three months of STN-DBS (*p* < 0.01). Other motor outcome data are shown in Table 2.

### 3.2. Behavior Rating Inventory of Executive Function-Adult Version

Compared to BRIEF-A normative data [22], the PD group preoperatively reported more pronounced difficulty in the domain of metacognitive executive function (MI) (*p* = 0.008). At subscale level, patients scored significantly higher than normative samples on Initiative (*p* = 0.004), Working Memory (*p* = 0.003), Plan/Organize (*p* = 0.004), and Task Monitor (*p* = 0.013).

After three months of STN-DBS, the BRIEF-A scores had declined significantly on the Global Executive Composite (GEC) (*p* = 0.011) and the Metacognition Index (MI) (*p* < 0.01). At subscale level, patients reported significantly less problems on the Initiate (*p* < 0.01), Plan/Organize (*p* < 0.01), and Task Monitor (*p* = 0.029) scales. There were no significant changes on the Behavioral Regulation Index (BRI) or its subscales. None of the patients had scores above the recommended clinical cut-off on any of the BRIEF-A validity scales at baseline or at three-month follow-up.

### 3.3. Symptom Checklist 90-Revised

At baseline, the PD patients reported greater burden of general psychiatric symptoms on the SCL-90-R Global Severity Index (GSI) than the age- and gender-matched normative Norwegian sample [24] (*p* < 0.01) and reported higher level of Somatization (*p* < 0.01), Obsessive-Compulsive symptoms (*p* < 0.01), Depression (*p* < 0.01), and Anxiety (*p* < 0.01). When gender differences were taken into account, men and women preoperatively reported the same level of psychiatric complaints on the SCL-90-R Global Symptom Index (0.5 ± 0.4). At subscale level, women and men in our patient group scored similar, except for the Somatization scale where women had higher scores than men (women; 1.0 ± 0.6, men; 0.8 ± 0.6) and the Hostility scale where women had lower scores then men (women; 0.0 ± 0.1 men; 0.2 ± 0.3). However, the skewed distribution of gender in the patient group makes statistical gender comparisons challenging.

At three-month follow-up, the mean scores of most of the SCL-90-R subscales remained the same as the preoperative scores. Scores on the Anxiety scale had, however, changed towards reduced anxiety (*p* = 0.038; Cohen's *d* = 0.4).

The SCL-90-R consists of three items regarding sleep disturbances: “trouble in falling asleep”, “restless or disturbed sleep,” and “early awakening.” We have performed exploratory subitems analyses, comparing the sum scores of these three sleep items before and after surgery. Paired sample *t*-tests showed that sum scores of these three items decreased significantly (0.6 ± 1.2, *p* = 0.006) from preoperative (1.8 ± 1.2) to three-month follow-up (1.0 ± 1.0), indicating improved sleep.

### 3.4. Apathy Evaluation Scale Self-Rated Version

Based on a cut-off score of 38 on the AES-S, the patients were not considered clinically apathetic neither before surgery (30.6 ± 5.9) nor at three-month follow-up (32.2 ± 6.6). At three-month follow-up, the AES-S sum score had increased towards more apathy, but not significantly (*p* = 0.16).

### 3.5. Correlations between the BRIEF-A, Motor Symptoms (MDS-UPDRS-III), the SCL-90-R, and the AES-S

There were no significant correlations between the BRIEF-A scores and the MDS-UPDRS-III scores before surgery or at three-month follow-up or to the difference in MDS-UPDRS off-scores between these two time-points.

Preoperatively, there were significant positive correlations between all three main indexes of the BRIEF-A and the Global Severity Index (GSI) of the SCL-90-R (GEC: *r* = 0.6, *p* < 0.01; MI: *r* = 0.6, *p* < 0.01; BRI: *r* = 0.6, *p* < 0.01), as well as with all the subscales of the SCL-90-R, with the exception of Somatization.

After three months of STN-DBS, there were significant positive correlations between the Global Executive Composite and the Metacognition Indexes of the BRIEF-A and the SCL-90-R subscale Obsessive-Compulsive (GEC: *r* = 0.4, *p* = 0.04; MI: *r* = 0.4, *p* = 0.04). The BRIEF-A Task Monitor subscale was positively correlated with SCL-90-R Depression scores (*r* = 0.4, *p* = 0.02). The AES-S was positively correlated with the BRIEF-A Global Executive Composite (GEC: *r* = 0.4, *p* = 0.012) and the Metacognition Index (MI: *r* = 0.5, *p* < 0.01).

### 3.6. Multiple Linear Regression Analysis

We performed stepwise linear regression to explore whether the elevated preoperative SCL-90-R scores (Global Severity Index (GSI), Somatization, Obsessive-Compulsive, Depression, and Anxiety) or changes in motor scores were possible predictors of the improvement observed in aspects of self-reported executive function after three months of STN-DBS. The significant changes in the BRIEF-A from baseline to 3-month follow-up in the Global Executive Composite (GEC), the Metacognition Index (MI), and the subscales Initiate, Plan/Organize, and Task Monitor were used as dependent variables. In the first step, the preoperative scores of the SCL-90-R Global Severity Index (GSI), Somatization, Obsessive-Compulsive, Depression, and Anxiety scales were used as independent variables.

In the next step, linear regression analysis was conducted with changes in the BRIEF-A scores used as dependent variables, and the differences between MDS-UPDRS-III off-scores from baseline to 3-month follow-up were used as independent variables.

Of all possible predictors, only the SCL-90-R preoperative Depression score was significantly associated with the change of the BRIEF-A Initiate subscale (*B* = −0.446, *p* = 0.05).

## 4. Discussion

Studies on the effect of STN-DBS on executive function in PD patients have reached different conclusions [15–18]. A limitation of many of these studies is the lack of documentation of how PD patients perceive their executive functioning in everyday life before and after the initiation of STN-DBS.

In this study we assessed whether there were differences in self-reported executive function in the everyday life of PD patients before and after three months of STN-DBS. We also examined whether patients' executive functioning was related to motor symptoms, psychiatric symptoms, or apathy.

We found that preoperatively the PD group reported higher levels of difficulties in everyday life executive function than normative samples [22]. The PD patients differed significantly on the Metacognition Index (MI) and its subscales Initiative, Working Memory, Plan/Organize, and Task Monitor. However, after three months of STN-DBS the patients reported significant improvement in their overall level of executive functioning, as reflected in the decreased score on the BRIEF-A Global Executive Composite. This indicates that after the initiation of STN-DBS, the patients subjectively experienced fewer executive problems overall and felt more capable in keeping up with everyday life demands. For example, they perceived less difficulty in actively coordinating and sustaining problem solving efforts to effectively complete tasks. At the subscale level, patients with PD reported significantly fewer problems on the Initiate, Plan/Organize, and Task Monitor scales. The Initiate subscale measures the ability to instigate involvement in a new activity without external prompting. The reduction in the Initiate subscale scores after three months of STN-DBS suggests that the patients experienced less difficulty in generating ideas or starting tasks. The lower scores on the Plan/Organize and Task Monitor subscales further indicate that they perceived improvement in their ability to actively set goals and organize information and action steps needed for task completion and monitoring of one's own performance [22]. However, no significant changes were seen in the BRIEF-A Behavioral Regulation Index. This index provides an overall measure of the ability to manage one's emotional and behavioral responses while engaged in daily activities, reflecting skills such as inhibitory control, shifting between activities, emotional control, and self-monitoring. Altogether, the BRIEF-A findings suggest that the improved executive functions were primarily related to metacognitive aspects of executive control and not behavioral aspects.

According to our findings, the level of motor symptoms was not associated with the self-reported executive functioning, neither preoperatively nor at three-month follow-up. Importantly, the significant improvement of motor symptoms observed after three months of STN-DBS did not have any predictive value on the self-reported improvement of executive function. These findings are in line with earlier work which found that neither clinical motor symptoms nor fine motor dexterity was related to reported executive dysfunction in individuals with PD [20]. However, some authors have suggested that there might be a relationship between cognition and motor dysfunction in PD patients and that executive dysfunctions are associated with bradykinesia [26]. Our findings also support the work of Wolz et al., 2012 [27], which found no correlations of severity changes of non-motor symptoms with motor improvement after STN-DBS.

Compared to the normative Norwegian population, the patient group experienced significantly more psychiatric symptoms preoperatively on both the SCL-90-R Global Severity Index (GSI) and the subscales Somatization, Obsessive-Compulsive symptoms, Depression, and Anxiety [24]. Notably, after initiation of STN-DBS, the mean scores of the SCL-90-R remained the same as before surgery, with exception of the Anxiety subscale score, which was significantly reduced. The dampened anxiety in our patient group is consistent with earlier findings of a large randomized multicenter study. That study found significant improvement in anxiety scores in PD patients with STN-DBS compared with the PD patients who received best medical treatment [28]. It is suggested that the improvement in anxiety is associated with the reduction of non-motor off-drug treatment periods [29]. However, the link between anxiety and DBS is yet to be confirmed (see Table 4).

Preoperatively, we found a significant positive correlation between the BRIEF-A Global Executive Composite and the Global Severity Index of the SCL-90-R. Similar associations between self-reported executive function and psychiatric complaints have previously been reported in clinical studies of other neurological patient groups [30]. High correlations between indexes of global psychological distress and global executive impairment suggest that they reflect overlapping aspects of psychological/behavioral functioning. In the present study, however, the correlation between the global severity indexes where no longer significant 3 months after the initiation of STN-DBS. Moreover, PD patients reported subjective improvement of their executive functioning, although the level of general psychiatric symptoms predominantly remained the same, with the exception of a reduction in anxiety.

Of all possible predictors, only preoperative Depression score of the SCL-90-R was found to have predictive value to the change of self-reported executive function. The level of depression prior to STN-DBS had negative predictive value for the improvement of the BRIEF-A subscale Initiate. As the Initiate scale reflects the ability to independently start a task or activity, it taps into the initiation problems typically observed in individuals with depressed mood. We found that the higher the level of depression before surgery, the less the change reported on the Initiate subscale after surgery. Depression might therefore prevent beneficial effect of STN-DBS in some aspects of how patients perceive their executive function. Still, the predictive capacity was modest, suggesting that other unmeasured neuropsychiatric symptoms or personal vulnerabilities such as impulsivity, mood swings, cognitive deficits, and lack of insight might interfere with self-reported executive function. Additionally, it cannot be ruled out that improvement of sleep after three months of STN-DBS might influence the patients self-report in daily life executive functioning, but this might also be an incidental finding. In a recent publication the authors studied the relationships among excessive daytime sleepiness, nighttime sleep quality, and cognitive impairment in PD. Daytime sleepiness in PD, but not nighttime sleep problems, was found to be associated with cognitive impairment in PD and (including executive function) in PD but, particularly, in demented PD patients [31].

In the current study, there was no significant change in apathy scores between baseline and at three-month follow-up. Our findings thus did not support other comparable short-term studies that showed significantly increased levels of apathy after three months of STN-DBS [13, 14]. However, in a previous study apathy occurred in over half of PD patients after a mean of 4.7 (range 2–12) months of STN-DBS. In that study the reduction of dopaminergic drugs was, however, more extreme than in most other published studies, including the present study. The authors suggested that apathy during STN-DBS mainly could be regarded a symptom of a delayed dopamine withdrawal syndrome [32].

As indicated earlier, apathy and executive dysfunction share common features. In this study, correlation analysis showed a weak, but significant, correlation between the AES-S and the BRIEF-A Global Executive Composite as well as with the Metacognition Index. Together with previously cited studies linking apathy to executive dysfunction [7, 8], these results suggest an association between self-rated apathy and self-reported executive functioning in everyday life.

### 4.1. Limitations

The sample size of this study was limited, although comparable to previous studies about the effects of STN-DBS in PD patients [13–15, 18]. With regard to the impact of STN-DBS on self-reported executive function on the BRIEF-A, there is a need to replicate findings in larger samples. A further limitation is that we did not attempt to correct for multiple comparisons. This implies that we accept the higher probability of type I errors. Without a treatment as usual control group we cannot rule out the possibility that patients who had been treated with dopaminergic medications alone would have shown the same changes in self-reported executive function as our STN-DBS group. However, obtaining a control group of PD patients that matches our group of PD STN-DBS patients is ethically challenging. One of the main reasons is that suitable PD patients would themselves be candidates for STN-DBS surgery. The follow-up period of this study was also relatively short. Future studies should examine whether there is a persistent trend of self-reported improvement in executive functioning. However, to enhance understanding of the changes in executive functions related to STN-DBS in PD, studies focusing on short-term changes are as relevant as studies about chronic STN-DBS state. This is due to the fact that chronic executive impairment occurring years after the initiation of STN-DBS might be explained by cognitive deterioration, which is part of the natural evolution of PD [33].

Future studies regarding the effects of STN-DBS on executive functioning in the everyday life of PD patients should preferably also include standardized neuropsychological tests assessing executive functioning. Additionally, cognitive deficits could interfere with self-assessed executive functioning. A lack of insight may be related to the degree of cognitive impairment [34], resulting in underreporting of cognitive symptoms. Informant-based assessment could provide a complementary approach. This would particularly be helpful in the event that the patients' cognitive status should worsen.

The range of the preoperative BRIEF-A mean *T*-scores in our study was from 46.6 (±8.0) to −55.9 (±11.3). According to the criteria for clinically significant score elevation (*T*-scores at or above 65) provided in the BRIEF-A manual, our patients do not fulfil the clinical criteria for significant executive dysfunction. However, our PD patients preoperatively reported a significantly higher score on the Metacognition Index compared to American normative data.

Moreover, previous studies of PD and other neurological pathologies have shown scores in the same range as our PD group. In the work of Lanni et al. [20] the range of self-reported mean *T*-scores on the BRIEF-A was from 51.1 (±14.6) to 53.6 (±14.8). In the study of Kudlicka et al. [21] PD patient with performance-based executive deficits reported a mean BRIEF-A *T*-score of 58.62 while PD patients without executive deficits reported a mean *T*-score of 55.95 on the Global Executive Composite Index. Lovstad et al. [30] studied executive functions in patients with focal orbital or lateral prefrontal lesions using the BRIEF-A. These patient groups showed mean *T*-scores in the same range as our PD patient group: GEC 51.4 (±9.3), MI 55.6 (±9.8), and BRI 51.6 (±10.3). In that study the mean *T*-scores of the Norwegian healthy control group were 0.5–1 SD below those of the American normative sample. BRIEF-A *T*-scores around 40 have also been reported in other studies including healthy Norwegian adults [35], suggesting that healthy Norwegians tend to report better executing functioning than their healthy American counterparts. Based on both our own findings and the findings of other studies, we therefore believe that *T*-scores below the clinical cut-off of 65 in Norwegian patient groups can provide important information about executive functioning in daily life. We recognize, however, that our PD group did not report profound executive impairments.

## 5. Conclusion

In the current study, patients with PD reported significant improvement in executive functioning in everyday life after three months of STN-DBS, both in the overall level of executive functioning as well as in specific domains such as initiating, planning/organizing of tasks, and task monitoring. In addition, anxiety significantly declined after three months of STN-DBS, while other psychiatric symptoms remained the same as preoperatively. There was no significant change in apathy scores after surgery. The improvement of self-reported executive functioning did not correlate with motor improvement after STN-DBS. Only preoperative depressed mood had predictive value to the improvement of executive function and appears to prevent potentially favorable outcomes from STN-DBS on some aspects of executive function. Depression in PD patients should therefore be treated prior to STN-DBS. The BRIEF-A might be assessing other aspects of executive function than performance-based measures and appears to be a useful tool to include in a broad assessment of executive functioning in everyday life. We therefore propose that PD patients being screened for STN-DBS surgery should be evaluated with regard to self-reported executive functioning. Complementary information about PD patients' executive functioning in everyday life might enhance further specific treatment planning of STN-DBS.

## Figures and Tables

**Table 1:** Demographic and disease characteristics of PD patients by the time of inclusion. IQ-score was estimated from the Wechsler Abbreviated Scale of Intelligence (WASI).

Gender	31 males/9 females
Age in years, mean (SD)	63.4 (6.4)
Duration of disease in years, mean (SD)	12.1 (3.8)
Years of education, mean (SD)	13.5 (3.4)
Estimated IQ, mean (SD)	102 (28.3)
Mattis DRS, mean (SD)	140.1 (3.3)

**Table 2:** Results of neurological measurements; Movement Disorder Society-Sponsored Revision of the Unified Parkinson's Disease Rating Scale (MDS-UPDRS-III) motor scores, Hoehn and Yahr disease stage, and levodopa equivalent daily dose (LEDD) in mg preoperatively and at 3-month follow-up.

	Preoperatively Mean (SD)	3-months follow-up Mean (SD)	Mean differences (SD)	*p* value
MDS-UPDRS III Off, mean (SD)	48.9 (12.4)	19.8 (9.7)	29.1 (10.7)	<0.001
MDS-UPDRS III On, mean (SD)	15.9 (9.4)	12.2 (7.8)	3.7 (9.0)	0.014
Hoehn and Yahr Off, mean (range)	3.1 (2–4)	2.2 (1–3)	0.9 (0.6)	<0.001
Hoehn and Yahr On, mean (range)	1.9 (0–3)	1.8 (0–3)	0.1 (0.5)	0.152
LEDD in mg, mean (SD)	1217 (433)	636 (327)	581 (368)	<0.001

**Table 3:** Comparison of the BRIEF-A general composite score (GEC), the two index scales (MI and BRI), and the nine clinical subscales (Inhibit, Shift, Emotional Control, Self-Monitor, Initiate, Working Memory, Plan/Organize, Task Monitor, and Organization of Materials), preoperatively and after 3 months of STN-DBS. Group mean *T*-scores and standard deviations (SD) for each scale are reported along with mean differences and *p* values. BRIEF-A validity scales are reported with mean raw scores and standard deviations (SD). ^*∗*^Bonferroni's adjusted *p* values.

BRIEF-A scales	Preoperative mean *T*-score (SD)	3-month follow-up mean *T*-score (SD)	Mean difference (SD)	*p* value	Cohen's effect size
Global Executive Composite (GEC)	53.0 (9.8)	49.3 (11.4)	3.6 (7.9)	0.011	0.5
Metacognition Index (MI)	55.0 (10.6)	50.7 (11.9)	4.3 (7.8)	0.002^*∗*^	0.6
Initiate	55.9 (11.3)	49.8 (11.3)	6.1 (8.8)	<0.001^*∗*^	0.7
Working Memory	55.4 (9.8)	53.3 (12.2)	2.1 (8.6)	0.161	
Plan/Organize	55.3 (10.0)	50.1 (10.5)	5.2 (9.0)	0.002^*∗*^	0.6
Task Monitor	54.4 (9.8)	50.1 (10.6)	4.3 (11.0)	0.029	0.4
Organization of Materials	51.4 (10.5)	50.4 (10.9)	1.0 (8.3)	0.482	
Behavioral Regulation Index (BRI)	49.8 (8.8)	47.5 (10.1)	2.3 (8.3)	0.114	
Inhibit	51.1 (7.9)	48.5 (7.9)	2.5 (8.6)	0.089	
Shift	51.4 (9.7)	49.7 (11.1)	1.7 (10.0)	0.319	
Emotional Control	49.2 (8.7)	47.5 (8.9)	1.7 (8.0)	0.230	
Self-Monitor	47.5 (8.2)	46.6 (8.0)	0.9 (8.4)	0.513	
Validity Scale	Raw score (SD)	Raw score (SD)			
Negativity	0.1 (0.3)	0.3 (0.6)			
Infrequency	0.8 (0.9)	1.1 (1.0)			
Inconsistency	2.9 (1.8)	2.9 (2.9)			

**Table 4:** Comparison of the SCL-90-R Global Severity Index (GSI) and the nine symptom dimensions between age- and gender-matched Norwegian normative population and patients' scores preoperatively and after three months of STN-DBS. Data are presented as mean-scores and standard deviations (SD) along with mean differences between patients' scores preoperatively and at three months follow-up and *p* values.

	Age- and gender- matched normative sample Mean (SD)	Preoperatively Mean (SD)	3-month follow-up Mean (SD)	Mean difference (SD)	*p* value
Global Severity Index (GSI)	0.3 (0.0)	0.5 (0.4)	0.4 (0.3)	0.1 (0.4)	0.119
Somatization (SOM) scale	0.4 (0.0)	0.8 (0.6)	0.7 (0.4)	0.2 (0.8)	0.203
Obsessive-Compulsive (O-C) scale	0.4 (0.0)	0.8 (0.6)	0.6 (0.5)	0.1 (0.7)	0.379
Interpersonal Sensitivity (I-S) scale	0.3 (0.0)	0.4 (0.5)	0.3 (0.3)	0.1 (0.5)	0.112
Depression (DEP) scale	0.3 (0.1)	0.7 (0.5)	0.5 (0.4)	0.1 (0.5)	0.224
Anxiety (ANX) scale	0.2 (0.0)	0.6 (0.5)	0.3 (0.3)	0.2 (0.6)	0.038
Hostility (HOS) scale	0.2 (0.0)	0.2 (0.3)	0.2 (0.2)	0.0 (0.3)	0.998
Phobic Anxiety (PHOB) scale	0.2 (0.0)	0.2 (0.5)	0.3 (0.4)	0.0 (0.6)	0.731
Paranoid Ideation (PAR) scale	0.2 (0.1)	0.2 (0.4)	0.2 (0.3)	0.0 (0.4)	0.539
Psychoticism (PSY) scale	0.2 (0.0)	0.2 (0.3)	0.1 (0.2)	0.1 (0.3)	0.209

## References

[1] Svenningsson P., Westman E., Ballard C., Aarsland D. (2012). Cognitive impairment in patients with Parkinson's disease: diagnosis, biomarkers, and treatment. *The Lancet Neurology*.

[2] Robbins T. W., Cools R. (2014). Cognitive deficits in Parkinson's disease: a cognitive neuroscience perspective. *Movement Disorders*.

[3] Cummings J. L. (1993). Frontal-subcortical circuits and human behavior. *Archives of Neurology*.

[4] Alvarez J. A., Emory E. (2006). Executive function and the frontal lobes: a meta-analytic review. *Neuropsychology Review*.

[5] Uekermann J., Daum I., Bielawski M. (2004). Differential executive control impairments in early Parkinson's disease. *Journal of Neural Transmission. Supplementum*.

[6] Santangelo G., Trojano L., Barone P., Errico D., Grossi D., Vitale C. (2013). Apathy in Parkinson's disease: diagnosis, neuropsychological correlates, pathophysiology and treatment. *Behavioural Neurology*.

[7] Butterfield L. C., Cimino C. R., Oelke L. E., Hauser R. A., Sanchez-Ramos J. (2010). The independent influence of apathy and depression on cognitive functioning in Parkinson's disease. *Neuropsychology*.

[8] Zgaljardic D. J., Borod J. C., Foldi N. S. (2007). Relationship between self-reported apathy and executive dysfunction in nondemented patients with Parkinson disease. *Cognitive and Behavioral Neurology*.

[9] Isella V., Melzi P., Grimaldi M. (2002). Clinical, neuropsychological, and morphometric correlates of apathy in Parkinson's disease. *Movement Disorders*.

[10] McKinlay A., Grace R. C., Dalrymple-Alford J. C., Anderson T., Fink J., Roger D. (2008). A profile of neuropsychiatric problems and their relationship to quality of life for Parkinson's disease patients without dementia. *Parkinsonism & Related Disorders*.

[11] Siri C., Cilia R., de Gaspari D. (2010). Psychiatric symptoms in Parkinson's disease assessed with the SCL-90R self-reported questionnaire. *Neurological Sciences*.

[12] Castrioto A., Lhommée E., Moro E., Krack P. (2014). Mood and behavioural effects of subthalamic stimulation in Parkinson's disease. *The Lancet Neurology*.

[13] Drapier D., Drapier S., Sauleau P. (2006). Does subthalamic nucleus stimulation induce apathy in Parkinson's disease?. *Journal of Neurology*.

[14] le Jeune F., Drapier D., Bourguignon A. (2009). Subthalamic nucleus stimulation in Parkinson disease induces apathy: a PET study. *Neurology*.

[15] Jahanshahi M., Ardouin C. M. A., Brown R. G. (2000). The impact of deep brain stimulation on executive function in Parkinson's disease. *Brain*.

[16] Pillon B., Ardouin C., Damier P. (2000). Neuropsychological changes between ‘off’ and ‘on’ STN or GPi stimulation in Parkinson's disease. *Neurology*.

[17] Smeding H. M. M., Speelman J. D., Koning-Haanstra M. (2006). Neuropsychological effects of bilateral STN stimulation in Parkinson disease: a controlled study. *Neurology*.

[18] Saint-Cyr J. A., Trépanier L. L., Kumar R., Lozano A. M., Lang A. E. (2000). Neuropsychological consequences of chronic bilateral stimulation of the subthalamic nucleus in Parkinson's disease. *Brain*.

[19] Pham U., Solbakk A. K., Skogseid I. M. (2015). Personality changes after deep brain stimulation in Parkinson's disease. *Parkinson's Disease*.

[20] Lanni K. E., Ross J. M., Higginson C. I. (2014). Perceived and performance-based executive dysfunction in Parkinson's disease. *Journal of Clinical and Experimental Neuropsychology*.

[21] Kudlicka A., Clare L., Hindle J. V. (2013). Awareness of executive deficits in people with Parkinson's disease. *Journal of the International Neuropsychological Society*.

[22] Roth R. M., Isquith P. K., Gioia G. A. (2005). *Behavioral Rating Inventory of Executive Function—Adult Version*.

[23] Derogatis L. R.

[24] Vassend O. I., Lian L., Andersen H. T. (1992). Norwegian versions of the NEO-personality inventory, symptom checklist 90 revised, and Giessen subjective complaints list. *Tidsskrift for Norsk Psykologforening*.

[25] Marin R. S., Biedrzycki R. C., Firinciogullari S. (1991). Reliability and validity of the apathy evaluation scale. *Psychiatry Research*.

[26] Domellöf M. E., Elgh E., Forsgren L. (2011). The relation between cognition and motor dysfunction in drug-naive newly diagnosed patients with Parkinson's disease. *Movement Disorders*.

[27] Wolz M., Hauschild J., Koy J. (2012). Immediate effects of deep brain stimulation of the subthalamic nucleus on nonmotor symptoms in Parkinson's disease. *Parkinsonism & Related Disorders*.

[28] Witt K., Daniels C., Reiff J. (2008). Neuropsychological and psychiatric changes after deep brain stimulation for Parkinson's disease: a randomised, multicentre study. *The Lancet Neurology*.

[29] Lhommée E., Klinger H., Thobois S. (2012). Subthalamic stimulation in Parkinson's disease: restoring the balance of motivated behaviours. *Brain*.

[30] Lovstad M., Funderud I., Endestad T. (2012). Executive functions after orbital or lateral prefrontal lesions: neuropsychological profiles and self-reported executive functions in everyday living. *Brain Injury*.

[31] Goldman J. G., Ghode R. A., Ouyang B., Bernard B., Goetz C. G., Stebbins G. T. (2013). Dissociations among daytime sleepiness, nighttime sleep, and cognitive status in Parkinson's disease. *Parkinsonism and Related Disorders*.

[32] Thobois S., Ardouin C., Lhommée E. (2010). Non-motor dopamine withdrawal syndrome after surgery for Parkinson's disease: predictors and underlying mesolimbic denervation. *Brain*.

[33] Krack P., Batir A., Van Blercom N. (2003). Five-year follow-up of bilateral stimulation of the subthalamic nucleus in advanced Parkinson's disease. *The New England Journal of Medicine*.

[34] Chatterjee A., Anderson K. E., Moskowitz C. B., Hauser W. A., Marder K. S. (2005). A comparison of self-report and caregiver assessment of depression, apathy, and irritability in Huntington's disease. *The Journal of Neuropsychiatry and Clinical Neurosciences*.

[35] Grane V. A., Endestad T., Pinto A. F., Solbakk A., Vaidya C. (2014). Attentional control and subjective executive function in treatment-naive adults with Attention Deficit Hyperactivity Disorder. *PLoS ONE*.

